# Multi-Color Two-Photon Microscopic Imaging Based on a Single-Wavelength Excitation

**DOI:** 10.3390/bios12050307

**Published:** 2022-05-06

**Authors:** Wei Yan, Yangrui Huang, Luwei Wang, Jin Li, Yong Guo, Zhigang Yang, Junle Qu

**Affiliations:** Key Laboratory of Optoelectronic Devices and Systems of Ministry of Education and Guangdong Province, College of Physics and Optoelectronic Engineering, Shenzhen University, Shenzhen 518060, China; weiyan@szu.edu.cn (W.Y.); 1900453023@email.szu.edu.cn (Y.H.); 1910454084@email.szu.edu.cn (J.L.); 1800284004@email.szu.edu.cn (Y.G.); zhgyang@szu.edu.cn (Z.Y.); jlqu@szu.edu.cn (J.Q.)

**Keywords:** fluorescence microscopy, two-photon microscopy, multi-color imaging, signal separation

## Abstract

Two-photon probes with broad absorption spectra are beneficial for multi-color two-photon microscopy imaging, which is one of the most powerful tools to study the dynamic processes of living cells. To achieve multi-color two-photon imaging, multiple lasers and detectors are usually required for excitation and signal collection, respectively. However, one makes the imaging system more complicated and costly. Here, we demonstrate a multi-color two-photon imaging method with a single-wavelength excitation by using a signal separation strategy. The method can effectively solve the problem of spectral crosstalk by selecting a suitable filter combination and applying image subtraction. The experimental results show that the two-color and three-color two-photon imaging are achieved with a single femtosecond laser. Furthermore, this method can also be combined with multi-photon imaging technology to reveal more information and interaction in thick biological tissues.

## 1. Introduction

Two-photon microscopy (TPM) has been widely used in the research of nerve [1,2], immunology [3,4], and disease detection [5,6] due to its advantages of deep imaging depth, intrinsic optical sectioning capabilities, and high resolutions in three-dimension imaging. With the development of medicine and biology, it has become more and more important to reveal the location and structure of different organelles in cells, as well as the interaction process between organelles, which makes multi-color two-photon microscopy study of deep tissue living cells important practical significance. Currently, multi-color two-photon microscopy imaging is a research hotspot in biomedical photonics, the reported multi-color two-photon microscopy mainly includes the following implementation methods. On one hand, multiple femtosecond lasers are used as excitation sources to excite different fluorescence probes, however, it will make the optical path of the imaging system complicated, expensive, and reduce the stability of the system [7,8,9]. On the other hand, a new type of pulsed laser can be developed as a light source, which can simultaneously excite a variety of fluorescent dyes. For example, the ultra-short laser pulse is coupled to the fiber, and the supercontinuum laser pulse is obtained as the excitation light source to achieve multi-color two-photon imaging. This method relies on the development of laser and optical fiber technology, and the cost of the optical system is not economical [10,11]. Multi-color two-photon imaging based on a single-wavelength excitation can effectively simplify the complexity and cost of the optical system. The key of this method is to solve the problem of spectral crosstalk in the imaging procession. In 2020, Ueki et al. proposed a multispectral approach to produce crosstalk-free images of fluorescence with overlapping spectra that cannot be separated by using band-pass filters [12].

Here, we propose a signal separation strategy to solve the signal crosstalk problem, which can achieve multi-color two-photon imaging based on a single-wavelength excitation with the advantage of convenient operation and lower budget. We demonstrated two-color and three-color imaging of fixed cells in the two-photon microscopic imaging system.

## 2. Materials and Methods

**Figure 1:**
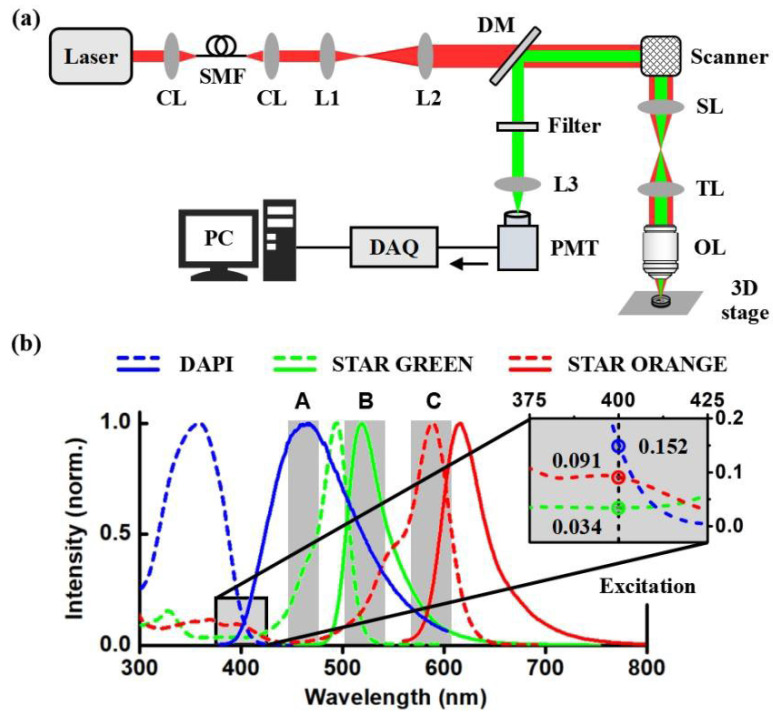
(**a**) Schematic of TPM. CL, collimating lens; SMF: single-mode fiber; L: lens; DM: dichroic mirror; SL: scan lens; TL: tube lens; OL: objective lens; PMT: photomultiplier tube; DAQ: data acquisition card. (**b**) The principle of the signals separation strategy. The dashed and solid lines represent the absorption and emission spectra of the fluorescent probes, respectively.

According to the literature reported [13], the quantum efficiency of fluorescence in multiphoton imaging was inversely proportional to the laser pulse width used for multiphoton excitation. Therefore, we adopted a femtosecond laser as the multiphoton excitation light source in this paper. As shown in Figure 1a, the excitation beam was provided by a pulsed Ti: sapphire laser (Chameleon Ultra II, Coherent, Santa Clara, CA, USA) that ran at 800 nm with a 140 fs pulse width and 80 MHz repetition frequency in the home-built two-photon microscope system. To optimize the wavefront of the laser spot, a 1 m long single-mode polarization-maintaining fiber (DH-FP780-FC-1, DHC, Thorlabs, Newton, MA, USA) was used, which can output the fundamental mode of laser with a Gaussian wavefront. The excitation beam was scanned by the X-Y scanning galvanometer (6210H, Cambridge Technology Inc., Cambridge, MA, USA) after passing through a dichroic mirror (DM). The combination of a scan lens (SL) and a tube lens (TL) is implemented after the scanner to expand the diameters of the laser beam to take full advantage of the numerical aperture of the objective. Samples are fixed on a three-dimensional stage (MPC-385 Series, Sutter Instrument Co., Novato, CA, USA). The fluorescence signal from the samples was collected and detected by a photomultiplier tube (H7422-40, Hamamatsu Photonics, Hamamatsu, Japan) after passing through a filter and a lens, To reduce costs, we set up a rotating wheel device in front of the detector to fix the band-pass filters. By rotating the wheel, different filters were selected to transmit the fluorescence signal at different wavelengths. Transmittance curves of different filters were shown in Figure S1.

The spectral signal separation method was performed with the same excitation wavelength to excite different probes and then used appropriate filters to select the fluorescence signals, which emitted from one probe or two probes. If the fluorescence signals were from two different probes (signal crosstalk), we could separate different fluorescent signals using a signals separation strategy similar to digital image processing, which is simple and convenient [14,15,16,17,18,19]. Our proposed multi-color two-photon microscopy imaging based on a single-wavelength excitation was realized with a home-built two-photon microscopy imaging system, suitable filters, and the signals separation strategy. The basic principle of the signal separation strategy was shown in Figure 1b. As a classic probe for labeling nucleus, DAPI can also be used in multi-photon imaging [20,21]. In addition, the commercial probes (STAR GREEN and STAR ORANGE, Abberior, Germary) commonly used in STED imaging have extremely resistance to bleaching and light stability, which can be used in two-photon imaging. Figure 1b shows the spectra of three fluorescence probes, which involve the absorption range of 300 nm to 600 nm (the dashed lines). To perform two-photon excitation on these three probes, an excitation wavelength needs to be determined. From the enlarged image in Figure 1b, one can be seen that the absorption coefficients of the three probes are 0.152, 0.091, and 0.034 at the wavelength of 400 nm, respectively. Therefore, we selected an 800 nm laser as the laser source for two-photon imaging, resulting in fluorescence signals with a similar intensity level. Since the emission spectra of DAPI and STAR GREEN are highly overlapped, it is impossible to separate the two fluorescence signals with filters. However, the fluorescence signals can be distinguished in the following steps. First, we chose three bandpass filters according to their emission spectra. The fluorescence signal of DAPI can be obtained through the filter A, obtaining the DAPI image with the intensity of *I*_DAPI_, whereas the fluorescence signals of both DAPI and STAR GREEN were obtained by using the filter B, inducing the image with the intensity of *I*_B_, where *I*_B_ = *I*_GREEN_ + *αI*_DAPI_. In addition, the filter C can pass through the fluorescence signals of three probes, generating the image with the intensity of *I*_C_, where *I*_C_ = *I*_ORANGE_ + *βI*_B_. The symbols *α* and *β* are difference coefficients, which are determined by the sum of rates of the emission spectrum under the corresponding filter, i. e., *α* = ∑*E*_B_(*λ*)/∑*E*_A_(*λ*), *β* = ∑*E*_C_(*λ*)/∑*E*_B_(*λ*), where *E* denotes the emission coefficient. Second, signal separation based on image subtraction is performed. The fluorescence signal of STAR GREEN can be separated by using the signal separation strategy: *I*_GREEN_ = *I*_B_ − *αI*_DAPI._ Similarly, we can obtain the fluorescence signal of STAR ORANGE by using *I*_ORANGE_ = *I*_C_ − *βI*_B._ Finally, a three-color image is obtained at a single wavelength excitation by superimposing the separated images of pseudo-colors.

## 3. Results and Discussion

To verify the feasibility of the signals separation strategy in a multi-color two-photon imaging system based on a single-wavelength excitation, a commercial multi-color labeled fixed cells samples were used in this paper. In this sample, different dyes were used to stain different cell organelles, and the corresponding relationship was as follows. Golgi-Abberior STAR ORANGE was used to stain the dictyosomes. The microtubules were stained with phalloidin-Abberior STAR GREEN. The nucleus was stained with DAPI.

According to the fluorescence spectrum characteristics of the dyes, a pulsed laser with a wavelength of 800 nm was used as an excitation beam in the experiment, and the multi-color labeled samples were performed imaging by a 100X objective lens. When the excitation light power in front of the objective lens port was 18 mW, three fluorescence signals could be excited simultaneously. The fluorescence signal was captured by PMT through a bandpass filter. Three bandpass filters (A: ET460/30m, B: ET520/40m, and C: ET585/40m) were selected to obtain different fluorescence signals.

**Figure 2:**
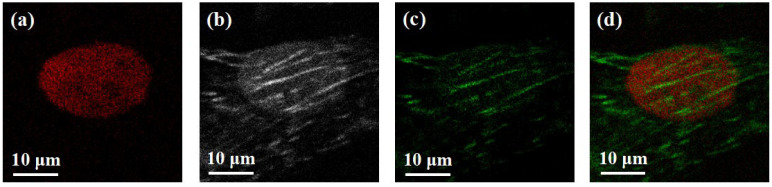
Two-color two-photon microscopic imaging.(**a**): nucleus; (**b**): nucleus and microtubules; (**c**): microtubules; (**d**): superimposed (**a**) and (**b**). Field of view (FOV): 40 μm, scale bar: 10 μm.

First, we performed a two-color two-photon microscopic imaging experiment to verify the method. The fluorescence signal of a single nucleus was captured by the PMT using filter A, and both cellular microtubules and nucleus fluorescence signals were obtained by using filter B, as shown in Figure 2a,b. We can see from Figure 2b that the nucleus and microtubules signals have crosstalk in the image. For the emission spectrum of the DAPI probe, the fluxes are 20.2 in filter A (integral value in the range of 445 nm to 475 nm) and 29.1 in filter B (integral value in the range of 500 nm to 540 nm). Therefore, the value of difference coefficient *α* is 0.7. Then, the intensity of Figure 2a was subtracted from Figure 2b by *α*-fold, and the microtubule image was obtained, which is shown in Figure 2c. Finally, we can see the relative positions of individual nucleus and microtubules by superimposing two images with different pseudo-colors (Figure 2d). For more data on two-color two-photon imaging, as shown in Supplementary Figure S2.

**Figure 3:**
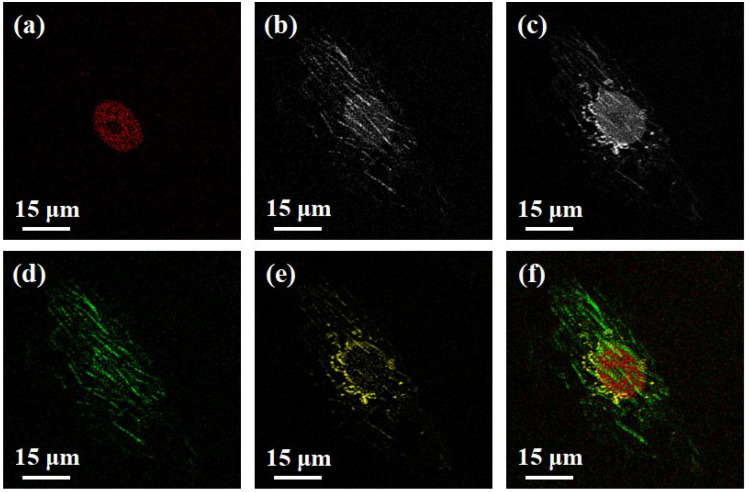
Three-color two-photon imaging. (**a**): nucleus; (**b**): nucleus and microtubules; (**c**): nucleus, microtubules and dictyosomes; (**d**): microtubules; (**e**): dictyosomes; (**f**): superimposed (**a**), (**d**) and (**e**). Field of view (FOV): 75 μm, scale bar: 15 μm.

Building on the foundation of two-color two-photon imaging, we further performed the effectiveness of the signal separation strategy to achieve three-color imaging using the fixed cells samples. Filter C was used to obtain the fluorescence signals of dictyosomes, microtubules, and the nucleus (Figure 3c). The flux in filter C is 4.3 for DAPI, inducing the difference coefficient *α* of 0.2. For the STAR GREEN probe, the fluxes in filter C and filter B are 5.9 and 31.7. Therefore, the value of the difference coefficient *β* is calculated to be 0.2. By using the signal separation strategy, the fluorescence signals of the dictyosomes (Figure 3e) and microtubules (Figure 3d) were separated. Finally, structures are distinguished by applying different pseudo-colors after image restoration, as shown in Figure 3f. The relative spatial position of each organelle can be seen intuitively, and the effective information is not lost compared with Figure 3c.

We confirmed a multi-color two-photon microscopic imaging method based on a single wavelength by using the signal separation strategy. However, we also note that there is a price for signal measurement and subtraction. Due to the limited bandwidth of the filters, the signal intensity is not as strong as in single-photon imaging. On the other hand, when the same filter passes through three or more fluorescence signals at the same time, it is easy to cause an incomplete signal separation. An effective way to solve this problem is to select filters that allow up to two kinds of signal crosstalk for each filter so that signals can be separated well by the signals separation strategy.

## 4. Conclusions

In this paper, a multi-color two-photon imaging method with a single-wavelength excitation by using a signal separation strategy was proposed. Two-color two-photon imaging of nucleus and microtubules, and three-color two-photon imaging of dictyosomes, microtubules, and nucleus were achieved through experiments. Experimental results show that this method can effectively solve the problem of signal crosstalk when each filter allows at most two kinds of signal crosstalk. The method is easy to be implemented on ordinary two-photon microscopy without additional optical elements, which has a low experimental system cost and simple post-processing. It can also be extended to polychromatic imaging of living cells.

## Data Availability

The data presented in this study are available on request from the corresponding author.

## Supplementary Materials

The following supporting information can be downloaded at: https://www.mdpi.com/article/10.3390/bios12050307/s1, Figure S1: Transmittance curves of different filters; Figure S2: Experimental results of two-color two-photon imaging.
