# Supplementary material for: Multi-Color Two-Photon Microscopic Imaging Based on a Single-Wavelength Excitation
Source: Biosensors (Basel). 2022 May 6;12(5):307. doi: 10.3390/bios12050307 (PMC9138471; doi:10.3390/bios12050307)
Supplement: Supplementary file 1 [file biosensors-12-00307-s001.zip › biosensors-1695252-supplementary.pdf]

biosensors

MDPI

Supplementary Materials

Multi-Color Two-Photon Microscopic Imaging Based on A Single-Wavelength Excitation

Wei Yan, Yangrui Huang, Luwei Wang *, Jin Li, Yong Guo, Zhigang Yang and Junle Qu

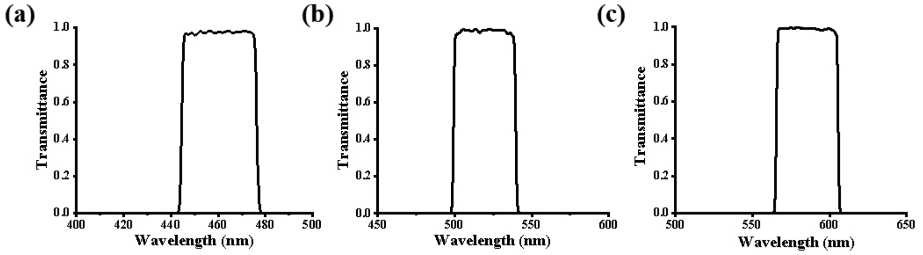

Figure S1. Transmittance curves of different filters. (**a**) ET460/30m; (**b**) ET520/40m; (**c**) ET585/40m.

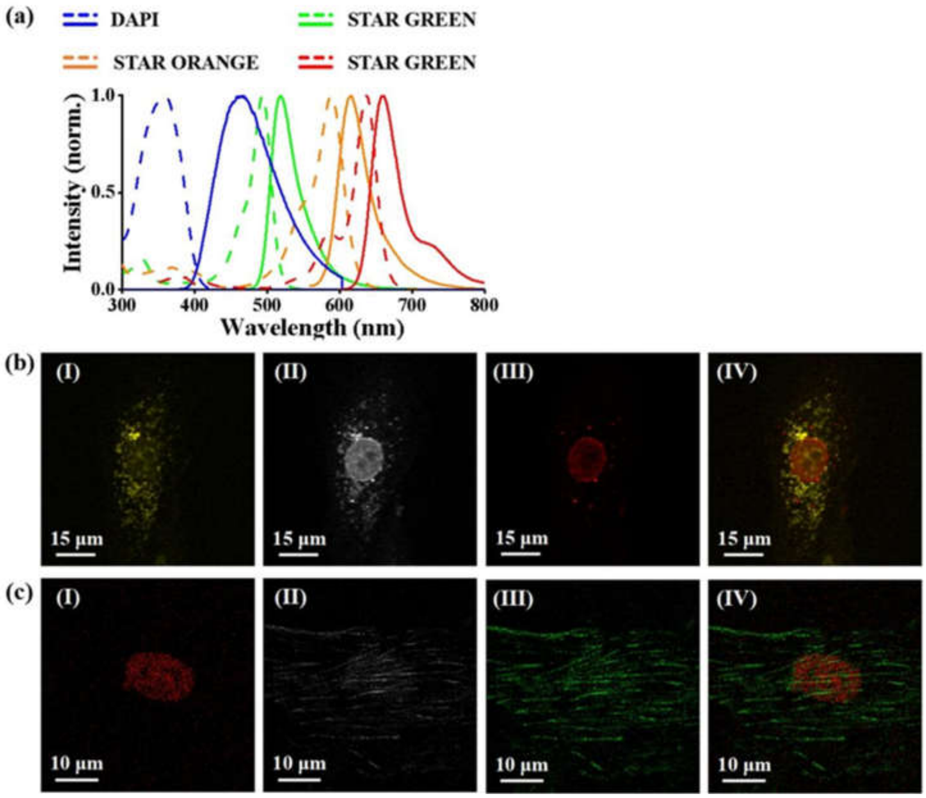

Figure S2. (**a**)The absorption and emission spectra of four fluorescent probes. (**b**)Two-color two-photon imaging of dictyosomes and nuclear pore complex. (I): dictyosomes; (II): dictyosomes and nuclear pore complex; (III): nuclear pore complex; (IV): superimposes (I) and (III). FOV: 80 μm, scale bar: 15 μm. (**c**) Two-color two-photon imaging of microtubules and nucleus. (I): nucleus; (II): nucleus and microtubules; (III): microtubules; (IV): superimposes (I) and (III). FOV: 40 μm, scale bar: 10 μm. Excitation wavelength of both (b) and (c) were 800 nm, excitation power in front of the objective lens was 20 mW. Excitation wavelength of both (b) and (c) were 800 nm, excitation power in front of the objective lens was 20 mW.
